# HIV-1 Replication in the Central Nervous System Occurs in Two Distinct Cell Types

**DOI:** 10.1371/journal.ppat.1002286

**Published:** 2011-10-06

**Authors:** Gretja Schnell, Sarah Joseph, Serena Spudich, Richard W. Price, Ronald Swanstrom

**Affiliations:** 1 Department of Microbiology and Immunology, University of North Carolina at Chapel Hill, School of Medicine, Chapel Hill, North Carolina, United States of America; 2 Lineberger Comprehensive Cancer Center, University of North Carolina at Chapel Hill, School of Medicine, Chapel Hill, North Carolina, United States of America; 3 Department of Neurology, University of California at San Francisco, San Francisco, California, United States of America; 4 UNC Center for AIDS Research, University of North Carolina at Chapel Hill, School of Medicine, Chapel Hill, North Carolina, United States of America; Duke University Medical Center, United States of America

## Abstract

Human immunodeficiency virus type 1 (HIV-1) infection of the central nervous system (CNS) can lead to the development of HIV-1-associated dementia (HAD). We examined the virological characteristics of HIV-1 in the cerebrospinal fluid (CSF) of HAD subjects to explore the association between independent viral replication in the CNS and the development of overt dementia. We found that genetically compartmentalized CCR5-tropic (R5) T cell-tropic and macrophage-tropic HIV-1 populations were independently detected in the CSF of subjects diagnosed with HIV-1-associated dementia. Macrophage-tropic HIV-1 populations were genetically diverse, representing established CNS infections, while R5 T cell-tropic HIV-1 populations were clonally amplified and associated with pleocytosis. R5 T cell-tropic viruses required high levels of surface CD4 to enter cells, and their presence was correlated with rapid decay of virus in the CSF with therapy initiation (similar to virus in the blood that is replicating in activated T cells). Macrophage-tropic viruses could enter cells with low levels of CD4, and their presence was correlated with slow decay of virus in the CSF, demonstrating a separate long-lived cell as the source of the virus. These studies demonstrate two distinct virological states inferred from the CSF virus in subjects diagnosed with HAD. Finally, macrophage-tropic viruses were largely restricted to the CNS/CSF compartment and not the blood, and in one case we were able to identify the macrophage-tropic lineage as a minor variant nearly two years before its expansion in the CNS. These results suggest that HIV-1 variants in CSF can provide information about viral replication and evolution in the CNS, events that are likely to play an important role in HIV-associated neurocognitive disorders.

## Author Summary

Human immunodeficiency virus type 1 (HIV-1) infection of the central nervous system (CNS) can lead to the development of a severe neurological disease termed HIV-1-associated dementia (HAD). Individuals diagnosed with HAD commonly have genetically distinct HIV-1 variants in their cerebrospinal fluid (CSF) that are not detected in the blood virus population, suggesting that independent viral replication is occurring in the CNS of HIV-1-infected subjects with severe neurological disease. We examined HIV-1 variants in the blood plasma and CSF of HAD subjects to determine the viral characteristics associated with the development of dementia during HIV-1 infection. We found that genetically distinct HIV-1 variants in the CSF of HAD subjects were either R5 T cell-tropic or macrophage-tropic. The R5 T cell-tropic viruses required high levels of the cellular surface receptor CD4 to enter cells, while macrophage-tropic viruses could enter cells with low levels of CD4, suggesting that HIV-1 can replicate in at least two cell types within the CNS during the course of dementia. Finally, macrophage-tropic viruses were detected in the CSF but poorly represented in the blood virus population. Our results suggest that HIV-1 variants in the CSF can provide information about independent viral replication in the CNS during the course of HIV-1 infection.

## Introduction

Human immunodeficiency virus type 1 (HIV-1) infects CD4^+^ T cells in the blood and lymphoid organs. In addition, infection of the central nervous system (CNS) can result in mild to severe neurological disease, including HIV-1-associated dementia (HAD) [1]. Although the incidence of HAD and minor cognitive motor disorder have been significantly reduced following the introduction of highly active antiretroviral therapy (HAART), these disorders continue to affect a substantial proportion of the HIV-1-infected population [2], [3]. The insufficient CNS penetration of some antiretroviral drugs or viral resistance may allow HIV-1 to persist in the CNS during the course of therapy [4], [5], [6], [7]. The success of HAART has led to an increased lifespan and an older demographic of HIV-infected subjects, and these subjects in particular have an increased risk of developing HAD due to their enhanced age [8], [9]. Less severe neurological problems associated with HIV-1 infection such as minor cognitive impairments may also be increasing [10], [11], indicating that neurological disorders will remain a problem for HIV-1-infected subjects in the future. Finally, unequal access to HAART and the potential of CNS involvement prior to the initiation of HAART makes the question of HIV replication in the CNS relevant to many infected people.

Several lines of evidence suggest that some HAD subjects can harbor macrophage-tropic HIV-1 variants [12], [13], [14], [15], [16], [17], a distinct phenotype associated with the ability to infect cells with low surface expression of CD4. The initiation of antiretroviral therapy results in rapid decay of virus in the blood, which is associated with virus replicating in activated CD4^+^ T cells [18], [19]; however, HIV-1 in the cerebrospinal fluid (CSF) can decay slowly with the initiation of therapy in some subjects with HAD, suggesting a longer-lived cell type as the origin of this virus [20], [21], [22]. Macrophage tropism does not appear to be a feature of the transmitted variants of HIV-1 [23], [24], leaving open the question of when and where macrophage-tropic variants of HIV arise and their role in HIV-1-associated pathogenesis.

Previous studies have reported that HIV-1 populations in the CSF of HAD subjects have increased viral genetic compartmentalization compared to virus in the blood [25], [26], and genetically distinct HIV-1 variants have been detected at autopsy in the CNS of some subjects with HAD [27], [28], [29], suggesting that autonomous viral replication is occurring in the CNS of subjects with more severe neurological disease. We examined HIV-1 variants in the CSF of HAD subjects to determine the viral genotypes and phenotypes associated with the development of HAD. Here we show that genetically compartmentalized CCR5-tropic (R5) T cell-tropic and macrophage-tropic HIV-1 populations are independently found in the CSF of subjects diagnosed with HIV-1-associated dementia. Our results demonstrate that HIV-1 can replicate in at least two cell types within the CNS in association with the development of dementia. Macrophage-tropic viruses were poorly represented in the blood population, highlighting the restricted, tissue-specific host range of these variants.

## Results

HIV-1 genetic compartmentalization and evolution of viral populations between the peripheral blood and CSF of HAD and non-HAD subjects were assessed using single genome amplification (SGA) of the viral *env* gene [30] and phylogenetic analyses (Table 1 and Table 2). Phenotypic characteristics of compartmentalized HIV-1 *env* genes from subjects with and without HIV-1-associated neurological disease were also measured to assess the entry phenotype of the compartmentalized virus. HIV-1 *env* genes were used to generate pseudotyped luciferase reporter viruses, which were then used to infect 293-Affinofile cells [31] with differential CD4 surface expression, and to infect monocyte-derived macrophages (MDM). Coreceptor tropism analysis revealed that most of the HIV-1 Env proteins were CCR5-tropic, including all of the Env proteins representing compartmentalized virus from the CSF (Table S1).

**Table 1:** Subject population clinical and virological characteristics.

		Cell counts (cells/µl)		HIV-1 RNA (log_10_ copies/ml)					
Subject ID	Sample date	CD4	CSF WBC^a^	Plasma	CSF	ADC Stage^b^	QNPZ-4^c^	Comp. virus phenotype^d^	CSF Decay^e^
4012	9/3/1997	295	16	5.18	4.39	0	−0.9	N/A	1.73
4030	9/16/1999	239	4	4.86	4.06	0	0.7	N/A	1.83
4021	11/5/1998	215	0	4.97	4.02	0	−0.2	N/A	1.64
4033	1/12/2000	173	28	4.83	5.23	2	−4.6	T-tropic	1.68
5003	11/3/1997	234	46	3.31	4.33	3	−4.0	T-tropic	1.21
7036	10/31/2002	327	20	4.67	3.89	0	−0.4	N/A	
	4/28/2003	324	30	4.97	4.41	0	−0.8	N/A	
	2/18/2004	267	240	5.12	5.37	2	−2.3	T-tropic	3.34
4051	8/20/2004	344	12	5.61	5.41	3	−6.4	M-tropic	2.39
4013	11/17/1997	148	10	4.62	4.75	1	−1.6	M-tropic	9.5
4059	8/16/2006	53	1	5.31	5.08	3	−6.7	M-tropic	9.42
5002	10/17/1997	59	4	4.24	5.32	3	−7.0	M-tropic	No decay
7115	7/8/2002	145	7	4.68	4.43	U	−1.5	M-tropic	
	12/3/2002	108	24	4.76	4.85	U	−1.5	M-tropic	
	4/8/2004	50	12	5.87	4.85	2	−6.2	M-tropic	
	5/11/2004	66	6	3.46	4.63	2	−8.4	M-tropic	13.58
	6/3/2004	150	7	2.97	3.54	3	−5.0	N/A	

a CSF white blood cell counts.

b AIDS dementia complex staging [1] conformed to the American Academy of Neurology criteria used at the time of diagnosis, and would also conform to current criteria for HAD diagnosis based on the degree of functional impairment in both cognitive and motor spheres. 0, neurologically asymptomatic; 1, mild neurological impairment; 2–3, moderate to severe HAD; U, uncertain ADC stage determination related to confounding conditions, see text for further discussion.

c Mean change from baseline (score of 0.0) of a four-item quantitative neurological performance battery score.

d Compartmentalized HIV-1 population phenotype. M-tropic, macrophage-tropic; T-tropic, R5 T cell-tropic; N/A, not applicable.

e Half-life in days of total viral RNA decay in the CSF after the initiation of antiretroviral therapy. The decay rate is listed next to the sample date that was closest to the date when therapy was initiated. Data summarized from ref. [20]. Subject antiretroviral drug regimens and CNS penetration effectiveness rank calculations for drug regimens, and CSF-compartmentalized variant decay calculations are described in detail in ref. [20].

**Table 2:** HIV-1 population characteristics in the CSF compartment.

		SGA amplicons				Slatkin-Maddison^c^		
Subject ID	Sample date	Plasma	CSF	% Comp. [95% CI]^a^	Comp. *env* avg. pairwise distance^b^	Migration events	P-value	CSF compartment^d^
4012	9/3/1997	24	21	19 [7–41]	N/A	13	0.1705	Eq
4030	9/16/1999	41	20	0 [0–19]	N/A	15	0.1516	Eq
4021	11/5/1998	27	16	43 [23–67]	0.016	8	0.002	Intermediate
4033	1/12/2000	22	22	64 [43–80]	0.007	9	0.0021	Comp, Amp
5003	11/3/1997	32	17	94 [71–99]	0.003	3	<0.0001	Comp, Amp
7036	10/31/2002	40	13	23 [7–51]	0.002	8	0.0049	Intermediate, Amp
	4/28/2003	38	30	0 [0–13]	N/A	23	0.7113	Eq
	2/18/2004	27	25	92 [74–99]	0.002	3	<0.0001	Comp, Amp
4051	8/20/2004	25	29	48 [31–66]	0.03	15	0.0908	Intermediate
4013	11/17/1997	33	24	83 [64–94]	0.023	5	<0.0001	Comp
4059	8/16/2006	27	20	97 [81–99]	0.029	2	<0.0001	Comp
5002	10/17/1997	21	20	90 [69–98]	0.017	3	<0.0001	Comp
7115	7/8/2002	35	25	44 [27–63]	0.002	14	0.0083	Amp
	12/3/2002	20	21	62 [41–79]	0.031	10	0.0256	Intermediate
	4/8/2004	22	19	21 [8–44]	N/A	13	0.3956	Eq
	5/11/2004	19	17	100 [78–100]	0.02	2	<0.0001	Comp
	6/3/2004	N/A	3	N/A	N/A	N/A	N/A	N/A

a The percent of CSF *env* sequences that were compartmentalized at each sampling time point. CSF *env* sequences were considered compartmentalized based on maximum-likelihood phylogenetic tree structures and strong bootstrap support. The 95% confidence intervals were calculated for the proportion of CSF-compartmentalized *env* sequences using the modified Wald method.

b The classification of clonal amplification was determined by calculating the average overall pairwise distance (nucleotide substitutions per site) between *env* sequences in the compartmentalized HIV-1 population. A CSF-compartmentalized HIV-1 population with an average pairwise distance <0.01 was considered to have clonal amplification. N/A, not applicable.

c The Slatkin-Maddison test for gene flow between populations was used to measure genetic compartmentalization between the blood plasma and CSF HIV-1 populations. Migration events describe the number of *env* migration events between the blood plasma and CSF HIV-1 populations for each phylogenetic tree. A P-value <0.05 indicates statistically significant genetic compartmentalization between the blood plasma and CSF HIV-1 populations.

d HIV-1 population characteristics in the CSF compartment. Eq, equilibration with the blood plasma; Intermediate, a subpopulation of the CSF is compartmentalized; Comp, significant compartmentalization in the CSF; Amp, clonal amplification of variants detected in the CSF; N/A, not applicable.

### HIV-1 variants in the CSF of neurologically asymptomatic subjects can be R5 T cell-tropic

**Figure 1:**
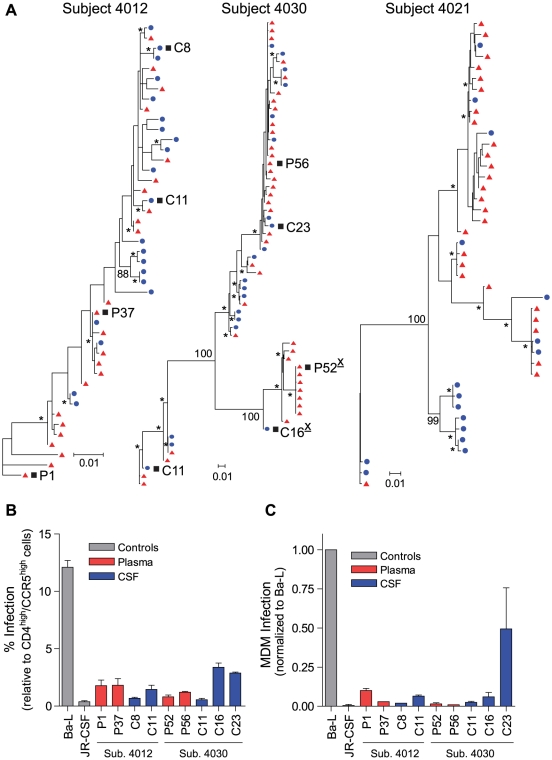
HIV-1 variants in the CSF of neurologically asymptomatic subjects can be R5 T cell-tropic. (A) Maximum-likelihood phylogenetic trees. *env* sequences from the CSF are labeled with solid blue circles, and plasma sequences are labeled with solid red triangles. Bootstrap numbers ≥70 are indicated with an asterisk at the appropriate nodes, and bootstrap values are listed at critical nodes in the trees. The genetic distance scale bar indicates the number of nucleotide substitutions per site between *env* sequences. HIV-1 *env* sequences selected for phenotypic analyses are indicated with a black square, and X4-tropic envelopes are indicated with an underlined, superscript “X” following the envelope name; all other envelopes were R5-tropic. (B) Single-cycle infection of HIV-1 Env-pseudotyped reporter viruses on CD4^low^CCR5^high^ 293-Affinofile cells. Receptor expression was measured as follows: CD4^low^ = 1,214 receptors/cell, CD4^high^ = 97,003 receptors/cell, CCR5^high^ = 34,431 receptors/cell. Data are expressed as means of quadruplicate wells ± s.d., and results are representative of two independent experiments. (C) Single-cycle infection of HIV-1 Env-pseudotyped reporter viruses on primary human MDM. Data shown are means of duplicate wells ± s.d. for one donor, and were normalized to infection of the control macrophage-tropic HIV-1 Ba-L envelope.

A range of genetic compartmentalization (0–43% of the total CSF sequences) was detected in the CSF HIV-1 populations of three neurologically asymptomatic subjects (Figure 1A and Table 2), which is consistent with reports from previous studies showing low but variable compartmentalization in asymptomatic subjects [26], [32]. Viruses pseudotyped with Env proteins derived from virus in the blood plasma and CSF of these neurologically asymptomatic subjects could only infect cells with high CD4 surface expression (Figure 1B; Table S1). In addition, most Env-pseudotyped viruses did not efficiently infect MDM (Figure 1C), although subject 4030 Env C23 infected about half as efficiently as the macrophage-tropic Ba-L envelope. This indicates that these viruses require the higher levels of surface CD4 found on activated CD4^+^ T cells for entry into target cells. While this represents a small sample size, this analysis demonstrates that T cell-tropic R5 viruses can be found in the CSF.

### Compartmentalized R5 T cell-tropic and macrophage-tropic HIV-1 populations are independently found in the CSF of subjects diagnosed with HIV-1-associated dementia

**Figure 2:**
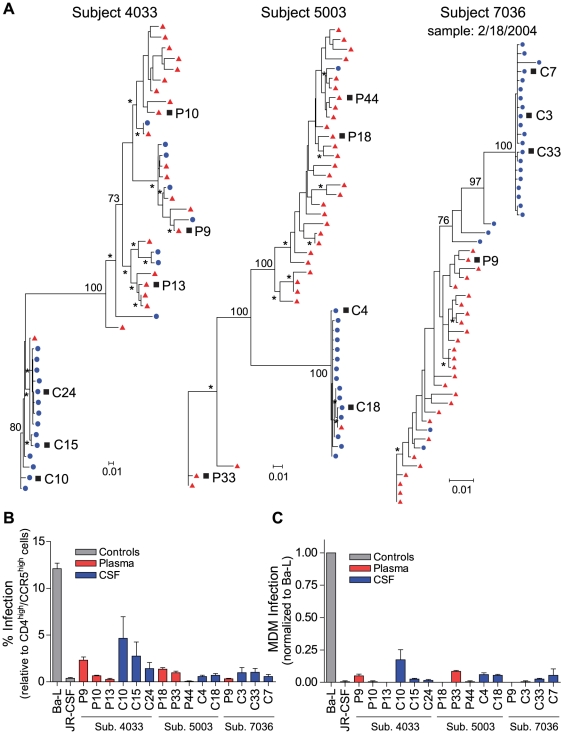
Compartmentalized R5 T cell-tropic HIV-1 populations are found in the CSF of subjects diagnosed with HAD. (A) Maximum-likelihood phylogenetic trees with compartmentalization and clonal amplification in the CSF. *env* sequences from the CSF are labeled with solid blue circles, and plasma sequences are labeled with solid red triangles. The phylogenetic tree characteristics are the same as those stated in Figure 1A. (B) Single-cycle infection of HIV-1 Env-pseudotyped reporter viruses on CD4^low^CCR5^high^ 293-Affinofile cells. Receptor expression was measured as follows: CD4^low^ = 1,214 receptors/cell, CD4^high^ = 97,003 receptors/cell, CCR5^high^ = 34,431 receptors/cell. Data are expressed as means of quadruplicate wells ± s.d., and results are representative of two independent experiments. (C) Single-cycle infection of HIV-1 Env-pseudotyped reporter viruses on primary human MDM. Data shown are means of duplicate wells ± s.d. for one donor, and were normalized to infection of the control macrophage-tropic HIV-1 Ba-L envelope.

**Figure 3:**
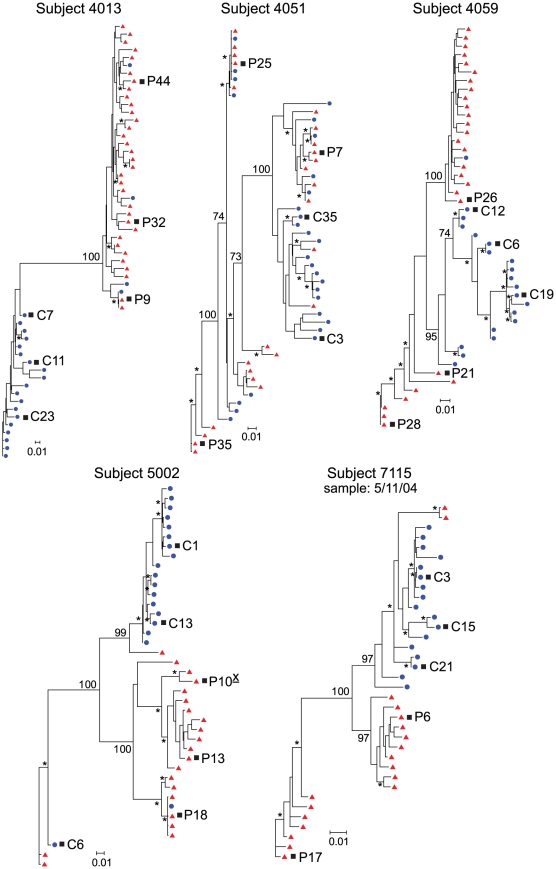
Compartmentalized HIV-1 populations in the CSF are associated with HAD. Maximum-likelihood phylogenetic trees are displayed for five HAD subjects with compartmentalization in the CSF. *env* sequences from the CSF are labeled with solid blue circles, and plasma sequences are labeled with solid red triangles. The phylogenetic tree characteristics are the same as those stated in Figure 1A.

Significant genetic compartmentalization was detected between the blood plasma and CSF HIV-1 populations of eight subjects diagnosed with HIV-1-associated neurological disease (Figure 2 and Figure 3; Table 2). We have previously shown that these subjects comprise two groups with respect to the rate of decay of compartmentalized virus in the CSF during the initiation of antiretroviral therapy: rapid decay in subjects 4033, 4051, 5003, 7036; and slow decay in subjects 4013, 4059, 5002, 7115 [20]. Phylogenetic analyses of the blood and CSF-derived virus in these subjects revealed significant compartmentalization and genetic distance between the blood plasma and CSF viral populations (Table 2; Figure 2A and Figure 3), indicating that sustained HIV-1 replication was likely occurring in the CNS of subjects with severe neurological disease. The detection of substantial compartmentalization in the HAD subjects was significant when compartmentalization versus an equilibrated population was compared to a model where each was equally likely (P = 0.03, Chi-squared test), or in comparison to the modest compartmentalization in the three asymptomatic subjects (P = 0.02, Fisher's Exact test).

Clonal amplification of a specific viral lineage was identified for CSF variants that were separated phylogenetically from the plasma virus population in three HAD subjects (subjects 4033, 5003, and 7036; Figure 2) who also had rapid decay of compartmentalized virus in the CSF [20]. The clonal amplification was seen as decreased *env* diversity in the CSF viral population, defined as having an average *env* pairwise distance <0.01 (see Table 2), and short branch lengths in the phylogenetic tree, both indicative of a recent expansion of a single variant. Clonal amplification of the CSF-compartmentalized HIV-1 population was associated with elevated CSF pleocytosis (Table 1; P = 0.036 using a two-tailed Mann-Whitney test). We found that the clonally amplified, compartmentalized HIV-1 variants were not able to infect cells expressing a low surface density of CD4 (Figure 2B) and did not efficiently infect MDM (Figure 2C), indicating that for these subjects the CSF-compartmentalized viruses were replicating in activated T cells.

**Figure 4:**
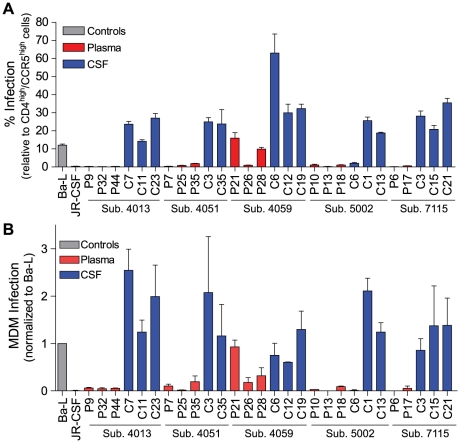
Compartmentalized HIV-1 populations in the CSF of some HAD subjects are macrophage-tropic. Phenotype data is displayed for HIV-1 *env* sequences from Figure 3. (A) Single-cycle infection of HIV-1 Env-pseudotyped reporter viruses on CD4^low^CCR5^high^ 293-Affinofile cells. Receptor expression was measured as follows: CD4^low^ = 1,214 receptors/cell, CD4^high^ = 97,003 receptors/cell, CCR5^high^ = 34,431 receptors/cell. Data are expressed as means of quadruplicate wells ± s.d., and results are representative of two independent experiments. (B) Single-cycle infection of HIV-1 Env-pseudotyped reporter viruses on primary human MDM. Data shown are means of duplicate wells ± s.d. for one donor, and were normalized to infection of the control macrophage-tropic HIV-1 Ba-L envelope.

Phylogenetic analysis showed significant compartmentalization between the blood plasma and CSF HIV-1 populations, and a more genetically complex viral CSF population, for the remaining five subjects with neurological disease (subjects 4013, 4051, 4059, 5002, and 7115; Figure 3). We demonstrated in a previous study that these subjects had slow decay of compartmentalized virus in the CSF after the initiation of antiretroviral therapy, although subject 4051 had rapid decay detected in the CSF due to a complex viral population and only partial compartmentalization of CSF virus [20]. Compartmentalized HIV-1 envelopes from the CSF of these five subjects efficiently infected cells with a low CD4 surface density (Figure 4A), and these Env proteins were macrophage-tropic based on their ability to infect MDM (Figure 4B). In contrast, most HIV-1 Env proteins derived from the blood of these subjects were not able to mediate infection of cells with low CD4 surface expression and could only infect cells with high CD4 levels (Figure 4), indicating adaptation for replication in activated T cells in the peripheral blood. However, a macrophage-tropic lineage was detected in the blood plasma of subject 4059 (Figure 3 and Figure 4), consistent with the previous observation that it is possible to isolate macrophage-tropic viruses from the blood of some subjects in late-stage disease [12], although this lineage was genetically distinct from that found in the CSF. The absence of the CNS macrophage-tropic virus lineage from the blood in the five subjects with macrophage-tropic virus in the CSF was statistically significant (P = 0.03, Chi-squared test). Finally, in the subjects with significant CSF compartmentalization there was a perfect correlation between the presence of R5 T cell-tropic virus and rapid viral load decay in the CSF, and with macrophage-tropic virus and slow viral decay in the CSF (P = 0.03, Fisher's Exact test).

### Macrophage-tropic HIV-1 variants in the CSF/CNS viral population can be detected prior to the development of overt, severe dementia

**Figure 5:**
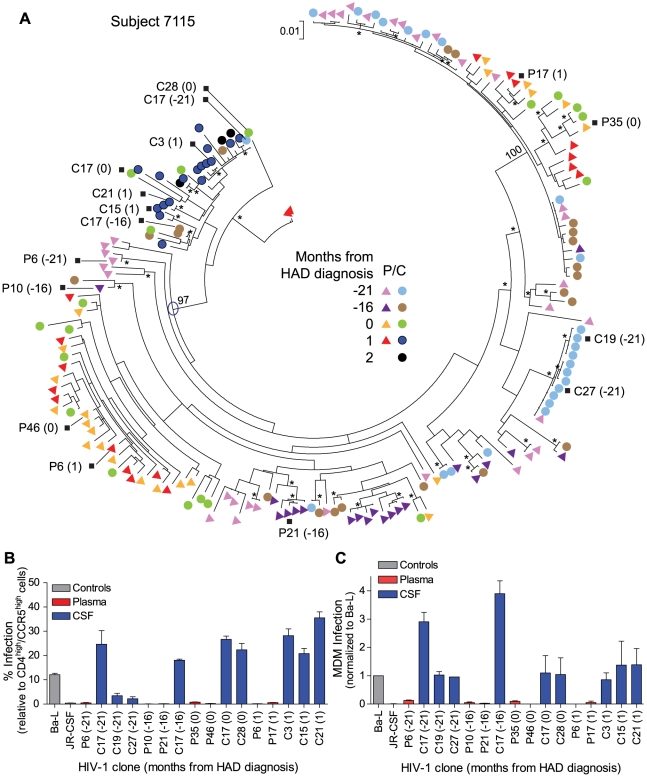
Macrophage-tropic HIV-1 variants in the CSF/CNS can be detected prior to the development of severe dementia. (A) Longitudinal phylogenetic analysis of *env* sequences from one subject with dementia. The genetic distance scale bar indicates the number of nucleotide substitutions per site between *env* sequences. HIV-1 *env* sequences selected for phenotypic analyses are indicated with a black square, and the node of divergence for the CSF M-tropic population is indicated with a blue open circle. Bootstrap numbers ≥70 are indicated with an asterisk at the appropriate nodes, and bootstrap values are listed at critical nodes in the tree. Months from HAD diagnosis correspond to the following sample dates: 7/8/2002 (−21), 12/3/2002 (−16), 4/8/2004 (0), 5/11/2004 (1), and 6/3/2004 (2). (B) Single-cycle infection of HIV-1 Env-pseudotyped reporter viruses on CD4^low^CCR5^high^ 293-Affinofile cells. Receptor expression was measured as follows: CD4^low^ = 1,214 receptors/cell, CD4^high^ = 97,003 receptors/cell, CCR5^high^ = 34,431 receptors/cell. Data are expressed as means of quadruplicate wells ± s.d., and results are representative of two independent experiments. (C) Single-cycle infection of HIV-1 Env-pseudotyped reporter viruses on primary human MDM. Data shown are means of duplicate wells ± s.d. for one donor, and were normalized to infection of the control macrophage-tropic HIV-1 Ba-L envelope.

We further examined macrophage-tropic and R5 T cell-tropic viral population dynamics in the CNS by conducting longitudinal *env* genotypic and phenotypic analyses for two subjects who progressed to HAD (Figure 5 and Figure S1). We detected a clonally amplified, compartmentalized R5 T cell-tropic population in the CSF of subject 7036 at the time of HAD diagnosis, but this population was not present in the CSF prior to the diagnosis of dementia (Figure S1), suggesting rapid expansion of a discrete viral population around the time of diagnosis. In addition, CSF viral populations at sampling time points prior to HAD diagnosis (10/31/2002 and 4/28/2003) were less compartmentalized, similar to CSF populations detected in neurologically asymptomatic subjects (Table 2). Clinical assessment also revealed a dramatic increase in CSF viral load and CSF neopterin, a pteridine associated with intrathecal immunoactivation [33], [34], over the study period (Table 1 and Figure S2A), which correlated with both the decline in neurological function from nearly asymptomatic to HAD (see QNPZ-4 scores in Table 1), and with the rapid expansion of a compartmentalized R5 T cell-tropic HIV-1 population in the CSF at this time-point.

The other subject (7115) had a compartmentalized macrophage-tropic HIV-1 population present in the CSF at the time of dementia diagnosis. We identified a macrophage-tropic lineage in the CSF of this subject (Figure 5A) spanning a period of approximately two years prior to the diagnosis of severe dementia [see clones C17 (−21) and C17 (−16)]. All of the HIV-1 *env* genes tested from this lineage encoded Env proteins that were macrophage-tropic based on the ability to infect cells with a low CD4 surface expression (Figure 5B), and the ability to infect MDM (Figure 5C). HIV-1 *env* genes from the blood populations at each time-point encoded proteins that were T cell-tropic. The viral sequences in the CSF from the macrophage-tropic lineage increased as a fraction of the population over time, especially between the time of HAD diagnosis and one month later. Neurological assessment of subject 7115 several years prior to HAD diagnosis revealed slightly impaired neurological performance (see QNPZ-4 scores in Table 1), and CSF viral load and neopterin were fairly stable over the two-year course of the study (Table 1 and Figure S2B), perhaps indicating earlier onset of mild neurological disease, although this subject had other confounding conditions including drug use and psychiatric disease. These confounding factors precluded an AIDS dementia complex (ADC) stage determination at earlier sampling time-points (7/8/2002 and 12/3/2002), although this subject was considered ADC stage 0 if this could be applied. Neurological performance markedly declined at the time of dementia diagnosis (QNPZ-4 of −6.2) and continued to decline thereafter. Taken together, our data indicate that in this subject macrophage-tropic HIV-1 variants existed as minor variants within a specific evolutionary lineage of the CSF/CNS viral population and eventually became the dominant CSF population, suggesting that confounding factors may have obscured an understanding of the potential earlier contribution of HIV-related CNS dysfunction. Neurocognitive impairment can result from multiple factors, and knowledge about viral replication in the CNS, as viewed through virus in the CSF, can provide insight about the potential contribution of HIV-1 to the neurological status of the patient.

## Discussion

We have demonstrated that genetically compartmentalized R5 T cell-tropic and macrophage-tropic HIV-1 populations are independently found in the CSF of subjects diagnosed with HIV-1-associated dementia. Our study was limited to a cohort of eight subjects with neurological disease who were receiving lumbar punctures at the time of HAD diagnosis and while initiating therapy, with samples available prior to therapy in two subjects followed longitudinally. In spite of a relatively small sample size we were able to link several features that distinguish two different virological states associated with severe neurological dysfunction. Compartmentalized HIV-1 populations in the CSF with R5 T cell-tropic entry phenotypes were separated phylogenetically from plasma virus populations, and were associated with clonal amplification of the CSF viral population. Replication in CD4^+^ T cells is consistent with both the rapid decay of compartmentalized virus in the CSF after the initiation of therapy in these subjects, and the migration of immune cells into the CNS/CSF as indicated by the presence of elevated CSF pleocytosis. Compartmentalized macrophage-tropic HIV-1 populations were associated with more genetically diverse viral populations in the CSF, and the slow decay of virus in the CSF after the initiation of therapy, indicative of viral replication in a long-lived cell. However, given the small sample size we cannot provide an accurate estimate of the relative frequency of each type of virologic state other than to note that they appeared with similar frequency in this cohort of eight subjects (three with compartmentalized R5 T cell-tropic virus, five with compartmentalized macrophage-tropic virus). Overall we detected a significantly compartmentalized CSF population in seven of these eight subjects suggesting that virus outgrowth in the CNS, whether macrophage-tropic or R5 T cell-tropic, will be a feature in a majority of HAD cases.

HIV-1 replication in the CNS is thought to occur in perivascular macrophages and/or microglia within the brain parenchyma [13], [35], [36]. We found that both R5 T cell-tropic and macrophage-tropic HIV-1 populations are independently associated with clinical dementia. This indicates a more complex interaction between HIV-1 and the CNS since the genetically compartmentalized R5 T-cell tropic viruses are unlikely to be replicating in macrophages or microglia given their requirement for high levels of CD4 to enter target cells. During simian immunodeficiency virus (SIV) infection of macaques, CNS infection is associated with the presence of infiltrating SIV-specific CD8^+^ T cells in the brain, but infiltrating CD4^+^ T cells have not been detected [37]. Trafficking of CD4^+^ T cells has been reported in the CNS during infection of other neurotropic viruses [38], [39]. We propose that the presence of viral antigen, especially during periods of increased HIV-1 replication in the CNS/CSF compartment, could drive the migration of both CD8^+^ and CD4^+^ T cells into the CNS/CSF (resulting in elevated CSF pleocytosis) and lead to persistence of compartmentalized virus through replication in the CD4^+^ T cells, and thus the apparent loss of this cell type. Consistent with the loss of these cells is the rapid decay of virus in the CSF during the initiation of therapy, which is considered a marker of viral replication in activated T cells [18], [19].

The pathological determinants of HAD are poorly understood. Some subjects with dementia exhibit HIV-1 encephalitis (HIVE) characterized by the presence of multinucleated giant cells of the macrophage/microglia origin and immunohistochemical evidence of viral replication [40], [41]. Although the incidence of HIVE has decreased during the HAART era, neuropathological changes in brain tissue, including glial activation and monocyte/macrophage infiltration [3], [42], [43], are still common. Future studies examining HIV-1 populations in paired blood, CSF, and brain tissue from HAD subjects with and without neuropathological findings will help determine whether there are physiological differences in brain pathology between subjects with R5 T cell-tropic versus macrophage-tropic HIV-1 variants as the predominant CSF population. Also, the appearance of macrophage-tropic viruses largely restricted to the CNS/CSF compartment is most consistent with the appearance of these viruses late in the infection time course of HIV-1, representing an expanded host range of the virus that is initially replicating in activated T cells. Although severe neurological disease associated with HIV-1 infection has declined in the HAART era, milder forms of neurological disease are increasing. In this study we detected a significantly compartmentalized macrophage-tropic HIV-1 population in the CSF of one subject with more mild neurological dysfunction (subject 4013; Table 1), illustrating the potential importance of understanding the correlates of HIV-1-associated neurological dysfunction with CNS/CSF viral population phenotypes.

HIV replication in the CNS can contribute to neurocognitive decline, so the ability to detect features of the CSF viral population associated with viral replication in the CNS may provide new opportunities to guide interventions prior to the development of overt neurological disease. In our study, one subject with longitudinal sampling (subject 7115) had macrophage-tropic variants present as a minor CSF population prior to the diagnosis of severe dementia (Figure 5). The application of sequencing technologies with greater capacity to sample a large number of viral genomes would allow the identification of minor CSF population variants, but this approach would rely on genotypic determinants of macrophage tropism rather than the phenotypic determinants used in our study. Several sequence determinants in *env* have been reported to be associated with macrophage tropism [27], [44], [45]; however, none of these determinants distinguishes the CSF-derived macrophage-tropic viruses from the paired blood-derived T cell-tropic viruses for the subjects in our study. Thus, the evolution of macrophage-tropic virus likely occurs through multiple pathways that will require a larger catalog of *env* sequences to allow reliable genotypic identification. It remains a possibility that the clonal amplification of R5 T cell-tropic viruses we detected in three HAD subjects is obscuring a smaller population of macrophage-tropic CNS virus, a question that could be addressed using more sensitive sampling methods. Developing a more complete understanding of the virological markers of CNS replication, and utilizing deep sequencing technologies to find minor populations, will provide opportunities to examine the use of CSF for information about viral replication in the CNS as a potential predictor of neurological involvement in the pathogenic process.

## Materials and Methods

### Ethics statement

This study was conducted according to the principles expressed in the Declaration of Helsinki. The studies were approved by the Committee for Human Research at the University of California at San Francisco, and written informed consent was obtained for the collection of samples from all subjects or their health care surrogates when informed consent was not considered possible.

### Study subject population

All subjects included in this study were HIV-1-infected individuals that eventually initiated highly-active antiretroviral therapy. The subject samples used for viral genetic compartmentalization and Env protein phenotypic analyses were collected during previous studies carried out at the University of California at San Francisco [46]. Serial blood plasma and cerebrospinal fluid (CSF) samples were collected from subjects at baseline prior to the start of therapy and during the initiation of antiretroviral therapy, and samples were collected longitudinally from subjects 7036 and 7115 for several years prior to the initiation of therapy. Plasma and CSF HIV-1 RNA concentrations were determined using the Amplicor HIV Monitor kit (Roche). Blood CD4 and CSF white blood cell (WBC) counts were performed by the San Francisco General Hospital Clinical Laboratory using routine methods. Subjects all underwent standardized neurological testing, including clinical criteria for diagnosis and staging of ADC. They also underwent brief quantitative neurological testing using four tests to derive a normalized score, the QNPZ-4 [47].

### Single genome amplification

HIV-1 RNA was isolated from blood plasma and CSF samples as previously described [20]. Viral RNA was reverse transcribed using Superscript III Reverse Transcriptase (Invitrogen) with oligo (dT) as the primer per the manufacturer's instructions. Single genome amplification of the full-length HIV-1 *env* gene through the 3′ U3 region was conducted as previously described [30]. Briefly, cDNA was diluted to endpoint, and nested PCR was conducted using the Platinum *Taq* High Fidelity polymerase (Invitrogen). The primers B5853 UP0 and LTR DN1, and B5957 UP1 and LTR DN1, were used for the first and second rounds of PCR, respectively [48]. The SGA amplicons were sequenced from the start of V1 through the ectodomain of gp41 [Hxb2 numbering of positions 6600–8000].

### Phylogenetic and compartmentalization analyses

Nucleotide sequences of the *env* genes were aligned using Clustal W [49], [50] or MAFFT software [51]. Sequences from each subject were codon aligned using GeneCutter (Los Alamos HIV-1 database; http://www.hiv.lanl.gov/content/sequence/GENE_CUTTER/cutter.html). Maximum likelihood phylogenetic trees were generated using PhyML [52] with the following parameters: HKY85 nucleotide substitution model, four substitution rate categories, estimation of the transition/transversion rate ratio, estimation of the proportion of invariant sites, and estimation of the gamma distribution parameter [53]. Compartmentalization of CSF viral populations by sequence was determined using the Slatkin-Maddison test for compartmentalization [54] by HyPhy software [55] using 10,000 permutations. Pairwise distance was calculated for HIV-1 *env* sequences in the CSF-compartmentalized population using MEGA 4.1 software [56], [57], [58].

### Construction of HIV-1 *env* clones

The SGA amplicons used in the cloning procedure were selected based on each subjects' phylogenetic tree structure and sequenced from the start of gp120 to the end of gp41. An additional PCR was conducted to amplify only the full-length HIV-1 *env* gene using the Phusion hot start high-fidelity DNA polymerase (Finnzymes) and the primers B5957F-TOPO (5′-CACCTTAGGCATCTCCTATGGCAGGAAGAAG-3′) and B8904R-TOPO (5′-GTCTCGAGATACTGCTCCCACCC-3′) following the manufacturer's instructions. HIV-1 *env* amplicons were then gel purified using the QIAquick gel extraction kit (Qiagen). The purified HIV-1 *env* genes were cloned into the pcDNA 3.1D/V5-His-TOPO expression vector (Invitrogen) using the pcDNA 3.1 directional TOPO expression kit (Invitrogen) and MAX Efficiency Stbl2 competent cells (Invitrogen) as per the manufacturer's instructions.

### Cells

293T and TZM-bl cells were cultured in Dulbecco's modified Eagle medium (DMEM) supplemented with 10% fetal bovine serum (FBS) and 100 µg/ml of penicillin and streptomycin. 293-Affinofile cells [31] were maintained in DMEM supplemented with 10% dialyzed FBS (12–14 kD dialyzed; Atlanta biologicals) and 50 µg/ml blasticidin (D10F/B). The Affinofile cell line was generously provided by Dr. Ben-Hur Lee. Monocyte-derived macrophages (MDM) were isolated from Ficoll-purified PBMCs (Biological Specialty Corporation, Colmar, PA) using the human monocyte enrichment kit without CD16 depletion (Stemcell Technologies) as per the manufacturer's instructions. Following isolation, MDM were seeded in 48-well tissue culture plates and cultured for 6 days. MDM were cultured in RPMI 1640 medium supplemented with 10% FBS, 100 µg/ml of penicillin and streptomycin, and 10 ng/ml recombinant human macrophage colony stimulating factor (M-CSF; Gibco).

### Env-pseudotyped viruses

Env-pseudotyped luciferase reporter viruses were generated by co-transfection of 293T cells with 3 µg HIV-1 *env* expression vector and 3 µg of the pNL4-3.LucR-E- plasmid (obtained from the NIH AIDS Research and Reference Reagent Program, Division of AIDS, NIAID, NIH) [59], [60] using the FuGENE 6 transfection reagent and protocol (Roche). Five hours post-transfection the medium was changed and the cells were incubated at 37°C for an additional 48 hours, and viral supernatants were harvested.

### Coreceptor tropism analysis

Two hours prior to infection the coreceptor inhibitors TAK-779 [61], [62] and bicyclam JM-2987 (hydrobromide salt of AMD-3100) [63], [64], [65] (both obtained from the NIH AIDS Research and Reference Reagent Program, Division of AIDS, NIAID, NIH) were added to TZM-bl cells at concentrations of 2.5 µM and 5 µM using the following conditions for each virus: no drug, TAK-779 only, AMD-3100 only, and both drugs. Cells were infected in the presence of drug using 50 µl of viral supernatant per well and spinoculated (2,000 rpm) for 2 hours at 37°C. Infections were incubated for 48 hours at 37°C, and then the cells were harvested and luciferase activity was assayed using the Luciferase assay system (Promega). All infections and conditions were conducted in triplicate.

### 293-Affinofile cellular surface expression of CD4 and CCR5

293-Affinofile cell [31] CD4 and CCR5 receptor expression was induced with tetracycline and ponasterone A (ponA; Invitrogen), respectively. Cells were induced for 18 hours at 37°C in a matrix format for a total of 24 induction levels with varying amounts of tetracycline (0–0.1 µg/ml) and ponA (0–2 µM/ml). Receptor expression was measured using quantitative fluorescence-activated cytometry (qFACS). Cells were stained with either phycoerythin (PE)-conjugated anti-human CD4 antibody (clone Q4120, BD Biosciences) or PE-conjugated mouse anti-human CCR5 antibody (clone 2D7, BD Biosciences). CD4 and CCR5 receptor levels were quantified using *QuantiBRITE* beads (BD Biosciences).

### Single-cycle infection of 293-Affinofile cells

Env-pseudotyped luciferase reporter viruses were initially titered on 293-Affinofile cells expressing the highest induction levels for CD4 (0.1 µg/ml tetracycline) and CCR5 (2 µM ponA) surface expression. The amount of pseudotyped virus used in the single-cycle Affinofile infection assay was normalized to 1×10^6^ relative light units for infection at the highest drug levels tested. All pseudotyped viruses were used within the linear range of the assay, and all infection conditions were assayed in quadruplicate.

Two days prior to infection, 96-well black tissue culture plates were coated with 10% poly-lysine in PBS and seeded with 293-Affinofile cells (2.5×10^4^ cells/well). Expression of CD4 and CCR5 was induced the following day by adding varying concentrations of tetracycline and ponasterone A as described above. Eighteen hours later, the induction medium was removed and fresh culture medium containing Env-pseudotyped virus was gently added to the cells. The infection plates were spinoculated [66] at 2,000 rpm for 2 hours at 37°C, and incubated for an additional 48 hours at 37°C. Infection medium was then removed and the cells harvested, and luciferase activity was assayed using the Luciferase assay system (Promega).

### Single-cycle infection of MDM

Env-pseudotyped luciferase reporter viruses were initially titered on 293-Affinofile cells expressing the highest induction levels for CD4 (0.1 µg/ml tetracycline) and CCR5 (2 µM ponA) surface expression. The amount of pseudotyped virus used in the single-cycle MDM infection assay was normalized to 1×10^7^ relative light units for infection at the highest 293-Affinofile drug levels tested. All pseudotyped viruses were used within the linear range of the assay, and all infection conditions for MDM were assayed in duplicate wells.

Env-pseudotyped virus was gently added to MDM in culture and the infection plates were spinoculated at 1,200×*g* for 2 hours at 37°C [66]. Unattached virus was removed from the MDM cultures by removing the medium gently without disturbing the cells. The MDM were then washed once with warm PBS supplemented with 1% fetal bovine serum to remove any residual virus, and once with warm RPMI 1640 medium supplemented with 10% fetal bovine serum and 100 µg/ml of penicillin and streptomycin to remove any remaining PBS/FBS. After the second wash, RPMI 1640 medium supplemented with 10% FBS, 100 µg/ml of penicillin and streptomycin, and 10 ng/ml M-CSF was added, and the cells were cultured for 5 days at 37°C. The culture medium was then removed and the cells were harvested and luciferase activity was assayed using the Luciferase assay system (Promega).

### Nucleotide sequence accession numbers

The HIV-1 *env* nucleotide sequences determined in this study have been deposited in GenBank under accession numbers JN562755-JN563605.

## Supporting Information

Figure S1 **Compartmentalized R5 T cell-tropic HIV-1 populations in the CSF appear concurrently with the development of dementia.** A maximum-likelihood phylogenetic tree is displayed containing three sampling times for subject 7036. Months from HAD diagnosis correspond to the following sample dates: 10/31/2002 (−16), 4/28/2003 (−10), and 2/18/2004 (0). The genetic distance scale bar indicates the number of nucleotide substitutions per site between *env* sequences. HIV-1 *env* sequences selected for phenotypic analyses are indicated with a black square.
(EPS)

Figure S2 **Longitudinal assessment of CSF viral load and CSF neopterin levels for subjects 7036 (A) and 7115 (B) prior to the diagnosis of HAD.** Red asterisks denote sample dates where HIV-1 population genotypes and phenotypes were analyzed, and the black arrow denotes the day of HAD diagnosis.
(EPS)

Table S1 **Phenotypic characteristics of HIV-1 Env-pseudotyped reporter viruses.**
(DOC)
