# Supplementary material for: HIV-1 Replication in the Central Nervous System Occurs in Two Distinct Cell Types
Source: PLoS Pathog. 2011 Oct 6;7(10):e1002286. doi: 10.1371/journal.ppat.1002286 (PMC3188520; doi:10.1371/journal.ppat.1002286)
Supplement: Table S1 — Phenotypic characteristics of HIV-1 Env-pseudotyped reporter viruses. (DOC) [file ppat.1002286.s003.doc]

**Table S1.** Phenotypic characteristics of Env-pseudotyped viruses.

|  |  |  |  |  | % Infection of 293-Affinofile cells | | |
| --- | --- | --- | --- | --- | --- | --- | --- |
| Subject ID | Sample date | Env clone | PSSM score^a^ | Coreceptor phenotype | CD4^high^/CCR5^high^ cells^b^ | CD4^low^/  CCR5^high^ cells^c^ | CD4^low^/  CCR5^low^ cells^d^ |
| Macrophage-tropic control |  | Ba-L | 0 | R5 | 100 | 12.1 | 10.6 |
| T cell-tropic control |  | JR-CSF | 0 | R5 | 100 | 0.4 | 0.4 |
| **4012** | 9/3/1997 | P1 | 0 | R5 | 100 | 1.8 | 1.4 |
|  |  | P37 | 0 | R5 | 100 | 1.8 | 1.8 |
|  |  | C8 | 0 | R5 | 100 | 0.7 | 0.4 |
|  |  | C11 | 0 | R5 | 100 | 1.5 | 1.3 |
| **4030** | 9/16/1999 | P52 | 1 | X4 | 100 | 0.8 | 1.3 |
|  |  | P56 | 0 | R5 | 100 | 1.2 | 1.2 |
|  |  | C11 | 0 | R5 | 100 | 0.5 | 0.6 |
|  |  | C16 | 1 | X4 | 100 | 3.4 | 4.8 |
|  |  | C23 | 0 | R5 | 100 | 2.9 | 2.8 |
| **4033** | 1/12/2000 | P9 | 0 | R5 | 100 | 2.3 | 2.2 |
|  |  | P10 | 0 | R5 | 100 | 0.6 | 0.6 |
|  |  | P13 | 0 | R5 | 100 | 0.2 | 0.2 |
|  |  | C10 | 0 | R5 | 100 | 4.6 | 2.3 |
|  |  | C15 | 0 | R5 | 100 | 2.7 | 1.4 |
|  |  | C24 | 0 | R5 | 100 | 1.5 | 0.8 |
| **5003** | 11/3/1997 | P18 | 0 | R5 | 100 | 1.3 | 1.0 |
|  |  | P33 | 0 | R5 | 100 | 1.0 | 0.8 |
|  |  | P44 | 0 | R5 | 100 | 0.1 | 0.0 |
|  |  | C4 | 0 | R5 | 100 | 0.6 | 0.5 |
|  |  | C18 | 0 | R5 | 100 | 0.7 | 0.5 |
| **7036** | 2/18/2004 | P9 | 0 | R5 | 100 | 0.3 | 0.2 |
|  |  | C3 | 0 | R5 | 100 | 1.0 | 0.5 |
|  |  | C7 | 0 | R5 | 100 | 1.0 | 0.5 |
|  |  | C33 | 0 | R5 | 100 | 0.6 | 0.3 |
| **4013** | 11/17/1997 | P9 | 0 | R5 | 100 | 0.3 | 0.3 |
|  |  | P32 | 0 | R5 | 100 | 0.2 | 0.2 |
|  |  | P44 | 0 | R5 | 100 | 0.3 | 0.4 |
|  |  | C7 | 0 | R5 | 100 | 23.7 | 19.7 |
|  |  | C11 | 0 | R5 | 100 | 14.2 | 11.9 |
|  |  | C23 | 0 | R5 | 100 | 27.0 | 20.8 |
| **4051** | 8/20/2004 | P7 | 0 | R5 | 100 | 0.4 | 0.2 |
|  |  | P25 | 0 | R5 | 100 | 0.8 | 0.3 |
|  |  | P35 | 0 | R5 | 100 | 1.9 | 1.1 |
|  |  | C3 | 0 | R5 | 100 | 25.0 | 15.1 |
|  |  | C35 | 0 | R5 | 100 | 23.8 | 14.5 |
| **4059** | 8/16/2006 | P21 | 0 | R5 | 100 | 16.0 | 12.8 |
|  |  | P26 | 0 | R5 | 100 | 0.9 | 0.6 |
|  |  | P28 | 0 | R5 | 100 | 9.9 | 5.7 |
|  |  | C6 | 0 | R5 | 100 | 63.1 | 22.6 |
|  |  | C12 | 0 | R5 | 100 | 30.0 | 16.7 |
|  |  | C19 | 0 | R5 | 100 | 32.3 | 23.7 |
| **5002** | 10/17/1997 | P10 | 1 | X4 | 100 | 1.1 | 1.6 |
|  |  | P13 | 0 | R5 | 100 | 0.2 | 0.5 |
|  |  | P18 | 0 | R5 | 100 | 1.1 | 1.0 |
|  |  | C1 | 0 | R5 | 100 | 25.6 | 18.8 |
|  |  | C13 | 0 | R5 | 100 | 18.8 | 13.2 |
|  |  | C6 | 0 | R5 | 100 | 2.1 | 1.5 |
| **7115** | 7/8/2002 | P6 | 0 | R5 | 100 | 0.5 | 0.4 |
|  |  | C17 | 0 | R5 | 100 | 24.7 | 17.3 |
|  |  | C19 | 0 | R5 | 100 | 3.4 | 2.0 |
|  |  | C27 | 0 | R5 | 100 | 2.2 | 1.5 |
|  | 12/3/2002 | P10 | 0 | R5 | 100 | 0.1 | 0.1 |
|  |  | P21 | 0 | R5 | 100 | 0.2 | 0.1 |
|  |  | C17 | 0 | R5 | 100 | 18.1 | 12.7 |
|  | 4/8/2004 | P35 | 0 | R5 | 100 | 0.8 | 0.7 |
|  |  | P46 | 0 | R5 | 100 | 0.2 | 0.2 |
|  |  | C17 | 0 | R5 | 100 | 26.7 | 19.6 |
|  |  | C28 | 0 | R5 | 100 | 22.3 | 16.1 |
|  | 5/11/2004 | P6 | 0 | R5 | 100 | 0.2 | 0.1 |
|  |  | P17 | 0 | R5 | 100 | 0.6 | 0.7 |
|  |  | C3 | 0 | R5 | 100 | 28.2 | 19.8 |
|  |  | C15 | 0 | R5 | 100 | 20.8 | 16.5 |
|  |  | C21 | 0 | R5 | 100 | 35.5 | 23.8 |

^a^Position-specific scoring matrix score (0 = R5-like V3 sequence, 1 = X4-like V3 sequence)

^b^CD4 density: 97,003 molecules/cell, CCR5 density: 34,431 molecules/cell

^c^CD4 density: 1,214 molecules/cell, CCR5 density: 34,431 molecules/cell

^d^CD4 density: 1,214 molecules/cell, CCR5 density: 2,101 molecules/cell
